# Differential Immunogenicity and Lung Disease-Inducing Potential of *Mycobacterium immunogenum* Genotypes and Impact of Co-Exposure with Pseudomonas: Optimizing a Mouse Model of Chronic Hypersensitivity Pneumonitis

**DOI:** 10.3390/ijms25042058

**Published:** 2024-02-08

**Authors:** Elisabet Johansson, Jagjit S. Yadav

**Affiliations:** Pulmonary Pathogenesis and Immunotoxicology Laboratory, Department of Environmental and Public Health Sciences, University of Cincinnati College of Medicine, Cincinnati, OH 45267-0056, USA

**Keywords:** *Mycobacterium immunogenum*, hypersensitivity pneumonitis, machine operator’s lung, immunogenicity, metalworking fluid, interstitial lung disease, *Pseudomonas fluorescens*

## Abstract

*Mycobacterium immunogenum* (MI) colonizing metalworking fluids (MWFs) has been associated with chronic hypersensitivity pneumonitis (HP) in machinists. However, it is etiologically unclear why only certain mycobacteria-contaminated fluids induce this interstitial lung disease. We hypothesized that this may be due to differential immunogenicity and the HP-inducing potential of MI strains/genotypes as well as the confounding effect of co-inhaled endotoxin-producers. To test this hypothesis, we optimized a chronic HP mouse model in terms of MI antigen dose, timepoint of sacrifice, and form of antigen (cell lysates vs. live cells) and compared six different field-isolated MI strains. Overall, MJY10 was identified as the most immunogenic and MJY4 (or MJY13) as the least immunogenic genotype based on lung pathoimmunological changes as well as Th1 cellular response (IFN-γ release). Infection with MI live cells induced a more severe phenotype than MI cell lysate. Co-exposure with *Pseudomonas fluorescens* caused a greater degree of lung innate immune response and granuloma formation but a diminished adaptive (Th1) immune response (IFN-γ) in the lung and spleen. In summary, this study led to the first demonstration of differential immunogenicity and the disease-inducing potential of field strains of MI and an interfering effect of the co-contaminating Pseudomonas. The improved chronic MI-HP mouse model and the identified polar pair of MI strains will facilitate future diagnostic and therapeutic research on this poorly understood environmental lung disease.

## 1. Introduction

Hypersensitivity pneumonitis (HP) is a diffuse interstitial lung disease (ILD) caused by inhalation of antigens composed of microbial (bacterial, fungal), avian, or mammalian proteins [1,2]. Traditionally, HP has been classified into chronic, subacute, and acute forms. Unlike the acute form, subacute and chronic HP appear more gradually upon repeated antigen exposure and are marked by cough, dyspnea, and, in the case of chronic HP, fatigue, weight loss [3,4], and even mortality [5]. The etiology and pathogenesis of HP are still poorly understood. It is estimated that only 1–15% of subjects exposed to the offending antigens develop HP, and a large number of those exposed become sensitized without progressing to overt disease [6]. HP can be difficult to diagnose, and differential diagnoses from other ILDs include sarcoidosis, non-specific interstitial pneumonia, and idiopathic pulmonary fibrosis [7,8]. Identifying an association between symptoms and exposure to a known HP antigen is a crucial step in the diagnostic process, which normally relies on radiography, lung function tests, and clinical symptoms.

Occupational HP in machinists, alternately called machine operator’s lung or metalworker’s lung [9], is associated with exposure to microbial-contaminated metalworking fluid (MWF) aerosols. Over the past three decades (since 1993), there have been several outbreaks of occupational HP among machinists/metalworkers exposed to MWF in the US [10,11,12,13,14,15,16,17,18,19,20,21] and Europe [22,23,24,25,26,27,28,29,30]. According to the reviews in 2016 [24] and 2021 [31], MWF exposure has become the most commonly recognized cause of occupational HP, having been rare before 2000. A 2017 epidemiology report [23] and a 2019 ILD specialists’ survey [32] concluded that over the past 20 years, exposure to MWFs has been the most frequently reported causative agent for occupational HP in the UK, with a 45% increase in MWF-associated HP cases during 1996–2015. In the US, the National Institute for Occupational Safety and Health (NIOSH) estimates that over 1.2 million machine workers are potentially exposed to MWF [18]. Additionally, this under-reported problem could potentially affect a larger population of workers, including those regularly exposed to MWF in other occupational settings. For instance, unaccounted metalworkers in low-tech shops continue to be at risk and are often outside of the HP tracking radar. Being a severely under-reported disease with relatively lesser-known epidemiology [23], there are no formal quantitative estimates of overall economic losses, including the burden of Disability Adjusted Life Years (DALYs) available.

Water-based MWF, the most used fluid type in today’s metal industries, often harbors mixed microbial communities composed of both Gram-positive and Gram-negative bacterial species [33,34,35,36]. Initially, members of the Gram-negative genus *Pseudomonas*, commonly reported in these fluids, were suggested as the etiological agents in machinists’ HP outbreaks [11,37]. Subsequently, HP outbreaks in metalworking plants were frequently associated with the presence of mycobacteria in MWF [10,15,30,38,39,40]. The most frequent mycobacterial species isolated from used MWFs and linked with outbreaks of HP was *Mycobacterium immunogenum* (abbreviated as MI), a member of the *M. chelonae–M. abscessus* (MCA) group of rapidly growing nontuberculous mycobacteria [39,40]. Since this first isolation and characterization from HP-linked metalworking fluids [39,40], *M. immunogenum* has been reported in other environmental sources, including drinking water, foods, and medical devices. Colonization and persistence in these environments and surfaces are due to its resistance to disinfectants/biocides [41,42,43] and potential to form biofilm [44]. In terms of pathogenicity, *M. immunogenum* has been linked with skin and soft tissue infections (SSTIs) [45,46], including cutaneous, pulmonary, eye, and other infections, and even brain abscess [47] as well as disseminated diseases such as septic shock [48]. To understand the host–pathogen interactions, our studies showed that *M. immunogenum* interacts with alveolar macrophages and activates stress-inducible MAP kinase-signaling pathways JNK and p38 to mediate inflammatory response [49]. The above developments in the biology and pathogenicity of this emerging mycobacterial pathogen have led to recent genome sequencing of some of its strains isolated from the above sources and infections [44,47,50].

Though initially a single genotype of *M. immunogenum* was claimed to dominate the industrial MWFs [51], our laboratory has since isolated and characterized several distinct genotypes (strains) of this species (*M. immunogenum*) from industry-wide screening of used MWFs using our customized molecular approaches [52,53]. However, the relative HP-inducing potential of the individual MI genotypes is not clear; differing immunogenic potential among the genotypes may help explain the variable appearance of HP symptoms in exposures involving mycobacteria-contaminated MWFs.

Understanding the pathogenesis mechanisms and treatment strategies of HP necessitates the use of experimental animal models, particularly considering the difficulty in obtaining timely access to the clinical cases of this occupational disease. Likewise, the lack of reliable laboratory diagnosis assays for HP warrants the use of experimental animal models to identify critical causal antigens that can differentiate symptomatic (HP) patients from the asymptomatic control (exposed control with no disease). Mouse models of HP have been developed primarily based on the thermoactinomycete *Saccharopolyspora rectivirgula* (SR), the antigen associated with Farmer’s HP. In those models, the Th1-biased mouse strain C57BL/6J is normally used, and the chronic exposure regimen involves nasal instillation of an antigen solution. Studies using those models have demonstrated features observed in clinical cases of Farmer’s HP, such as granuloma formation, lymphocyte infiltration, lung fibrosis, and release of the proinflammatory cytokine TNFα [54,55,56]. Past studies have employed similar exposure regimens in mouse challenge studies of machine operator’s lung (machinist’s HP) using *M. immunogenum* as the antigen [57,58]. In those studies, the sacrifice was performed 96 h after the instillation of the final dose, at which point most immuno–pathological responses could have partly or wholly subsided. Furthermore, those studies relied either via the intranasal route of exposure [58,59] that may often deposit inconsistently and only part of the actually instilled dose, or via the intratracheal route [57], which may prove more invasive for a repeated exposure regimen and routine investigations.

In this current study, we took a closer look at the *M. immunogenum* HP mouse model to understand and optimize the route of exposure, antigen dose, form of antigen, timepoint of sacrifice, and role of co-exposure to the most common Gram-negative bacterial co-contaminant *Pseudomonas* prevalent in MWF. The mouse model was used to compare immunogenicity and the disease-inducing potential of the six *M. immunogenum* genotypes (strains) isolated from field-drawn MWFs in our prior work [53]; the aim was to identify differentially immunogenic strains (to eventually identify HP-causal antigens) as well as understand the variability in the occurrence of HP cases from exposure to mycobacteria-contaminated MWFs. This study led to an optimized mouse model of MWF-associated mycobacterial HP and the selection of an immunogenically polar pair of *M. immunogenum* genotypes/strains (hyper- and hypo-immunogenic), critically important for future studies on the development of diagnostic tools and therapeutic targets.

## 2. Results

### 2.1. Optimization of Antigen Dose

This part of the study was designed as a screening experiment for selecting a dose of mycobacterial protein that provided a clear immuno–pathological response without being lethal or causing necrotizing lung damage. The response to the amount of mycobacterial protein for each instillation was studied over the dose range of 0 to 30 µg using the machinist’s HP-linked strain *M. immunogenum* 700506.

**Figure 1:**
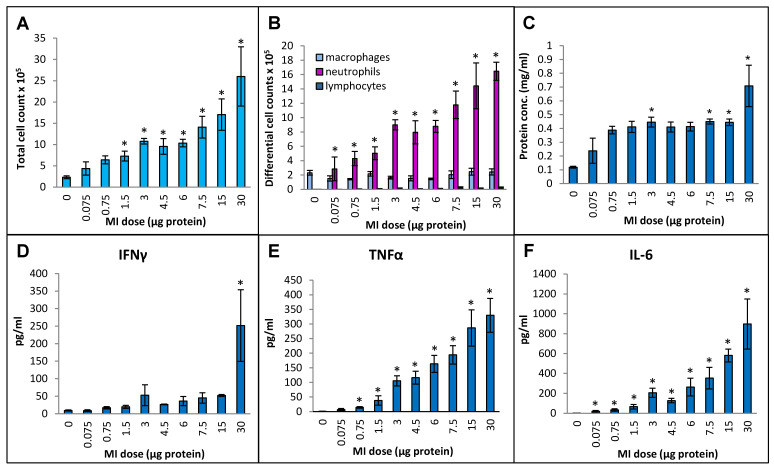
Dose–response relationship for induction of lung immunological response at increasing doses (0 to 30 µg protein/instillation) of *M. immunogenum* 700506 (designated as “MI” in the graphs) cell lysate in a chronic HP mouse model. Five mice were used for each treatment group. The chronic dose regimen was composed of repeated doses administered via oropharyngeal aspiration for three consecutive days a week for three weeks, and the response was measured in the bronchoalveolar lavage (BAL) fluid at 4 h post-instillation of the last dose in terms of the following parameters. Panels: (**A**) Total cell count. (**B**) Differential cell count. (**C**) Total protein concentration. (**D**) IFN-γ. (**E**) TNF-α. (**F**) IL-6. * Significantly elevated above control.

Cellular infiltration into the lung measured in terms of the total number of immune cells in bronchoalveolar lavage (BAL) fluid increased continuously by more than 10-fold (from 4.6 × 10^5^ ± 0.7 × 10^5^ cells/mL in the PBS-instilled control animals to 52 × 10^5^ ± 14 × 10^5^ cell/mL at the 30 µg dose) over the dose range tested (Figure 1A). Differential cell counting showed that the increase was due to a strong influx of neutrophils, and no significant increases in the number of macrophages or lymphocytes were seen (Figure 1B). The protein concentration in BAL fluid increased linearly during the initial part of the dose range (from 0.12 mg/mL at the 0 dose in control animals to 0.39 mg/mL at the 0.75 µg dose (Figure 1C). With subsequent increments in the dose, the protein concentration remained constant (~0.4 mg/mL) up to the 15 µg dose, followed by a notable increase (to 0.71 mg/mL) at the 30 µg dose. In terms of inflammatory cytokines, BAL fluid IFN-γ levels in the highest dose, 30 µg, showed a dramatic increase over the other doses (Figure 1D), which showed very mild increases that were not statistically significant. TNFα levels were gradually and significantly increased from 14 pg/mL BAL fluid at a dose of 0.75 µg to 330 pg/mL at a dose of 30 µg (Figure 1E). IL-6 levels were significantly increased from 19 pg/mL at 0.075 µg dose to 897 pg/mL at 30 µg dose (Figure 1F). Levels of IL-10 did not rise above the background levels over the tested dose range.

**Figure 2:**
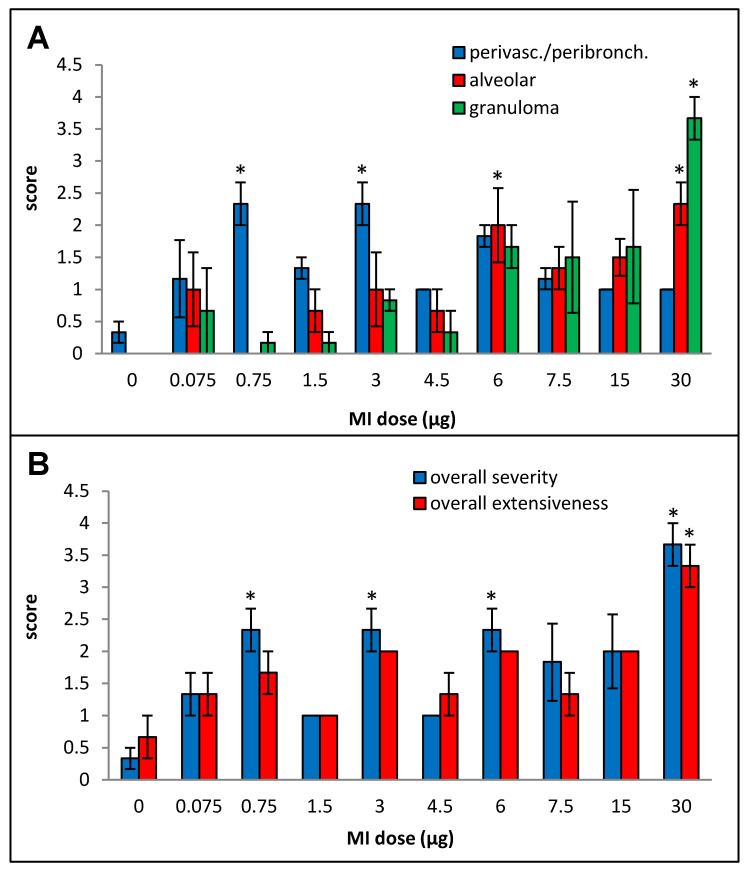
Dose–response relationship for histopathological changes caused by increasing doses (0 to 30 µg protein/instillation) of *M. immunogenum* 700506 (“MI”) cell lysate in a chronic exposure mouse model. Five mice were used for each treatment group. The chronic exposure regimen was composed of repeated doses administered via oropharyngeal aspiration for three consecutive days a week for three weeks and the histopathological response was assessed by H&E staining of the lung tissue at 4 h post-instillation of the last dose, scoring on an arbitrary scale from 0 to 4 for the following parameters. Panels: (**A**) Perivascular/peribronchiolar infiltration, alveolar infiltration, and granuloma formation. (**B**) Overall severity and extensiveness of the pathological changes. * Significantly elevated above control.

Primarily lymphocytic perivascular and peribronchial infiltration was observed at all doses, but the increase was statistically significant over the control animals only for doses 0.75, 3, and 6 µg, and no dose–response relationship could be derived (Figure 2A). Alveolar infiltration, mainly by neutrophils and macrophages, and granuloma formation were increased when compared to the background beginning at the 6 µg dose, but, due to inter-animal variations, the increases were statistically significant only at the 30 µg dose. At the highest dose (30 µg), significant necrotizing in the granulomas was observed.

### 2.2. Optimization of Timepoint for Sacrifice after the Final Dose Instillation

**Figure 3:**
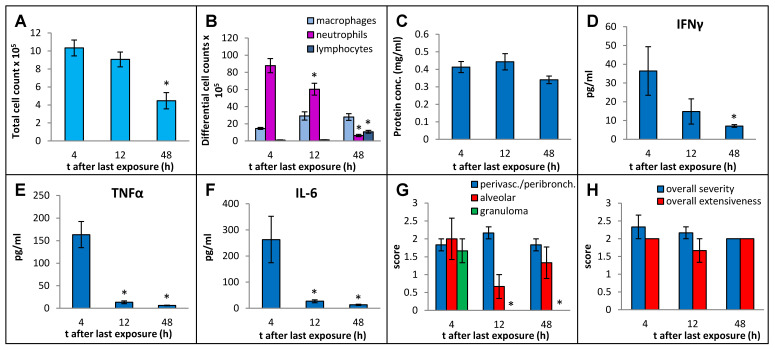
Effect of post-exposure time before sampling on immuno–pathological changes in the mouse lung due to oropharyngeal exposure to the whole cell lysate (10 µg protein/instillation) of *M. immunogenum* 700506 in a chronic exposure mouse model. The number of mice used for different treatment groups varied because of unanticipated losses and were as follows: five for 4 h timepoint and three each for 12 h and 48 h timepoints. BAL fluid and lung tissue harvested at different timepoints (4 h, 12 h, and 48 h) post-instillation of the last dose were analyzed for the following changes. Panels (**A**) through (**C**): Cellular and biochemical changes in BAL fluid, including (**A**) total cell count of immune cells; (**B**) differential cell count of immune cells; and (**C**) total protein content. Panels (**D**) through (**F**): Levels of different cytokines in BAL fluid, including (**D**) IFN-γ; (**E**) TNF-α; (**F**) IL-6.Panels (**G**,**H**): Pathological changes in the lung tissue scored on an arbitrary scale from 0 to 4, including (**G**) perivascular/peribronchiolar infiltration, alveolar infiltration, and granuloma formation; and (**H**) overall severity and extensiveness. * Significantly lower compared to the 4 h group.

In order to select an optimal post-exposure timepoint for the sacrifice that best captured the immunopathological response, we sacrificed the mice at 4 h, 12 h, and 48 h after final instillation in the 3-week exposure regimen, using MI 700506 at a dose of 6 µg mycobacterial protein (which corresponded to 2.5 × 10^7^ colony-forming units (cfu)). Whereas the total number of immune cells in BAL fluid decreased to about half (from 10.3 × 10^6^ at 4 h to 4.5 × 10^6^ at 48 h) (Figure 3A), the protein concentration in BAL fluid remained elevated without any significant decrease over this time range (Figure 3C). The strong influx of neutrophils abated gradually from 88 × 10^5^ cells/mL at 4 h to 6.3 × 10 × 10^5^ cells/mL at 48 h (Figure 3B). There was a mild but significant increase in macrophages at 12 h after the final instillation and the elevated level remained unaltered at 48 h. The number of lymphocytes in the BAL fluid was strongly increased at 48 h and constituted 24% of the total number of immune cells in the BAL fluid. Levels of IFN-γ, TNFα, and IL-6 in BAL fluid all decreased rapidly after the initial increase at 4 h and were down to background levels at the 48 h timepoint (Figure 3D–F).

Whereas perivascular/peribronchial and alveolar infiltration remained elevated throughout the experiment without any significant changes, granuloma formation was only observed at the 4 h timepoint post-exposure (Figure 3G). The overall severity and extensiveness of lung lesions remained unchanged (Figure 3H).

### 2.3. Dose–Response Relationship Using Live Cells of MI 700506

**Figure 4:**
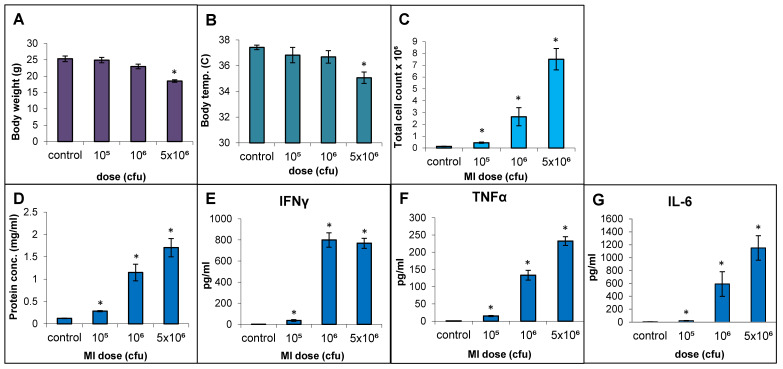
Effect of live bacterial cell suspension of *M. immunogenum* 700506 (“MI”) on dose-dependent induction of lung pathophysiological and immunological responses in a chronic exposure mouse model. Five mice were used for each treatment group. The chronic dose regimen was administered by repeated instillation of a defined dose (cfu/lung) via oropharyngeal aspiration for 3 consecutive days a week for 3 weeks and the response was measured at 4 h post-instillation of the last dose in terms of the following parameters. Whole body indicators: (**A**) Body weight. (**B**) Body temperature. BAL fluid parameters: (**C**) Total cell count. (**D**) Total protein concentration. (**E**) IFN-γ. (**F**) TNF-α. (**G**) IL-6. * Significantly different from control.

**Figure 5:**
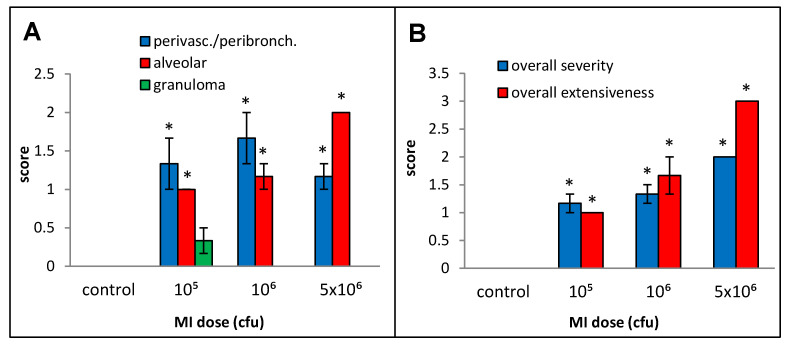
Effect of live bacterial cell suspension of *M. immunogenum* 700506 (“MI”) on lung histopathological changes in the chronic exposure mouse model. Increasing doses (cfu/animal) of monodispersed MI cell suspension were administered using oropharyngeal route of exposure and a chronic regimen as described in the caption of Figure 1. Five mice were used for each treatment group. The pathological changes in the lung were measured by scores for specific histopathological changes, including perivascular/peribronchiolar infiltration, alveolar infiltration, and granuloma formation (**A**), as well as overall scores for severity and extensiveness of damage (**B**), assigned on an arbitrary scale from 0 to 4. * Significantly elevated above control.

The responses to live cell suspension of MI 700506 (delivered oropharyngeally) in C57BL/6J mice were studied for doses of 10^5^, 10^6^, and 5 × 10^6^ cfu/animal, which corresponded to 0.024 µg, 0.24 µg, and 1.2 µg total mycobacterial protein, respectively. Mice were sacrificed 4 h after final dose instillation in the 3-week exposure regimen. The mean body weight (25.3 g) and mean body temperature (37.4 °C) showed a non-significant decline with time except for the highest dose, which showed a significant decrease (Figure 4A) to an extent reported in severe TB infection mouse models [60]. The total number of immune cells, protein concentration, IL-6 levels, and TNFα levels in BAL fluid were all significantly and dose-dependently increased for the three doses. IFN-γ levels in BAL fluid were strongly increased, with no significant difference between the two highest doses (Figure 4F). While perivascular/peribronchial and alveolar infiltrations were mild to moderate at all three doses, no significant granuloma formation was observed (Figure 5A). The overall extensiveness of the lung lesions increased from mild at the 10^5^ cfu dose to severe at the 5 × 10^6^ cfu dose (Figure 5B). Taken together, a comparison within the equivalent dose range tested for MI cell lysates and MI live cells revealed that the latter (cell suspensions) elicited a generally more severe phenotype.

### 2.4. Comparative Immunopathological Responses to Different Genotypes of M. immunogenum

**Figure 6:**
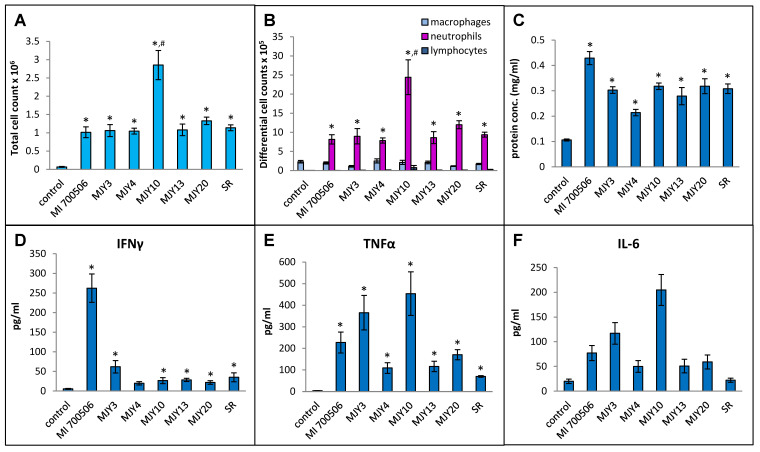
Comparison of six individual *M. immunogenum* genotypes for their potential to induce lung immunological changes in the chronic exposure mouse model. Five mice were used for each treatment group. The chronic exposure regimen was based on oropharyngeal exposure to *M. immunogenum* whole cell lysate at a repeated dose of 10 µg protein/instillation. BAL fluid was analyzed for the following parameters. Panels: (**A**) Total cell count. (**B**) Differential cell count. (**C**) Protein concentration. (**D**) IFN-γ. (**E**) TNF-α. (**F**) IL-6. The six MI genotypes compared were MI 700506, MJY3, MJY4, MJY10, MJY13, and MJY20. The actinomycete *S. rectivirgula* (SR) lysate (250 µg/animal) was used as positive control for comparison studies. Vehicle (PBS)-treated animals served as negative control. * Significantly elevated above control. ^#^ Cell counts were significantly higher in MJY10 than in all other genotypes.

Six previously characterized genotypes of *M. immunogenum* were compared for their ability to induce an HP-like phenotype in C57BL/6J mice, with *S. rectivirgula* serving as a positive control. Our working hypothesis was that the genomic differences in genotypes would manifest as differing antigenicity of gene products and differences in the ability to induce the HP-like phenotype. The dose chosen for this experiment was 10 µg mycobacterial protein (oropharyngeally administered as whole cell lysate), which provided a clear response in a dose optimization experiment using the strain MI 700506 without causing any necrotizing lung tissue. To capture the early immunopathological responses, a timepoint for sacrifice of 4 h after the final dose instillation optimized in the preceding part of the study was chosen. Body weights at the time of sacrifice were slightly lower in the mice exposed to mycobacterial protein lysates compared to controls and mice in the *S. rectivirgula* group, but the differences were not statistically significant. Total cell counts in BAL fluid were significantly higher in all exposed mice compared to the control group, and the counts in the MJY10 group were significantly higher than all other genotype groups, including the *S. rectivirgula* group (Figure 6A). The total number of macrophages in BAL fluid was not significantly elevated in any of the exposed groups, and there was no significant difference between the groups. The total number of neutrophils was strongly increased in all exposed groups, with significantly higher counts in the MJY10 group compared to all other groups (Figure 6B). The lymphocyte counts were too low to yield any meaningful comparison. Protein levels in BAL fluid were significantly increased in all exposed groups relative to controls, with the greatest increase in the MI 700506 group, showing significant differences from other genotypes except MJY10 and MJY20 (Figure 6C). The only group that had significantly elevated levels of IFN-γ in BAL fluid was the MI 700506 group (Figure 6D), while IL-10 and IL-12 levels were below the limits of detection for all genotypes. TNFα and IL-6 were at the highest levels in the MJY10 group, followed by the MJY6 and MI 700506 groups, and the lowest levels were in the MJY4 and MJY13 groups (Figure 6E,F). Levels of IL-4 were below the limit of detection for all genotypes as well as for *S. rectivirgula*-exposed mice.

**Figure 7:**
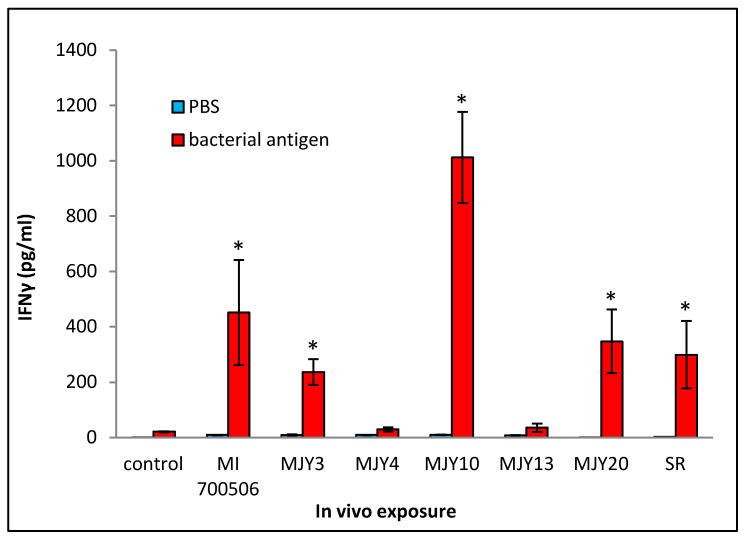
Comparison of individual *M. immunogenum* genotypes for their potential to induce T-cell response (in terms of release of IFN-γ) in the sensitized splenocytes isolated from the corresponding chronic exposure mouse model. The chronic exposure regimen was based on administration of a repeated dose of whole cell lysate at 10 µg protein/instillation. The sensitized splenocytes were isolated from the genotype-challenged mice and rechallenged with the same MI genotype cell lysate, as described under Materials and Methods section (Section 4). The six *M. immunogenum* genotypes (MI 700506, MJY3, MJY4, MJY10, MJY13, and MJY20) and the positive (SR) and negative (vehicle) controls for comparison were the same as described in the caption of Figure 6. * Significantly elevated above control.

Cultured splenocytes from each mouse were rechallenged with the respective antigen originally used for the in vivo exposure, and the induction of IFN-γ release was measured. Following the same trend as that of most of the BAL fluid parameters, the rechallenge of splenocytes from mice exposed to MJY10 resulted in the highest IFN-γ induction, whereas MJY4 and MJY13 rechallenged splenocytes yielded the weakest responses (Figure 7). Levels of IL-4 were not above the background for any of the genotypes.

**Figure 8:**
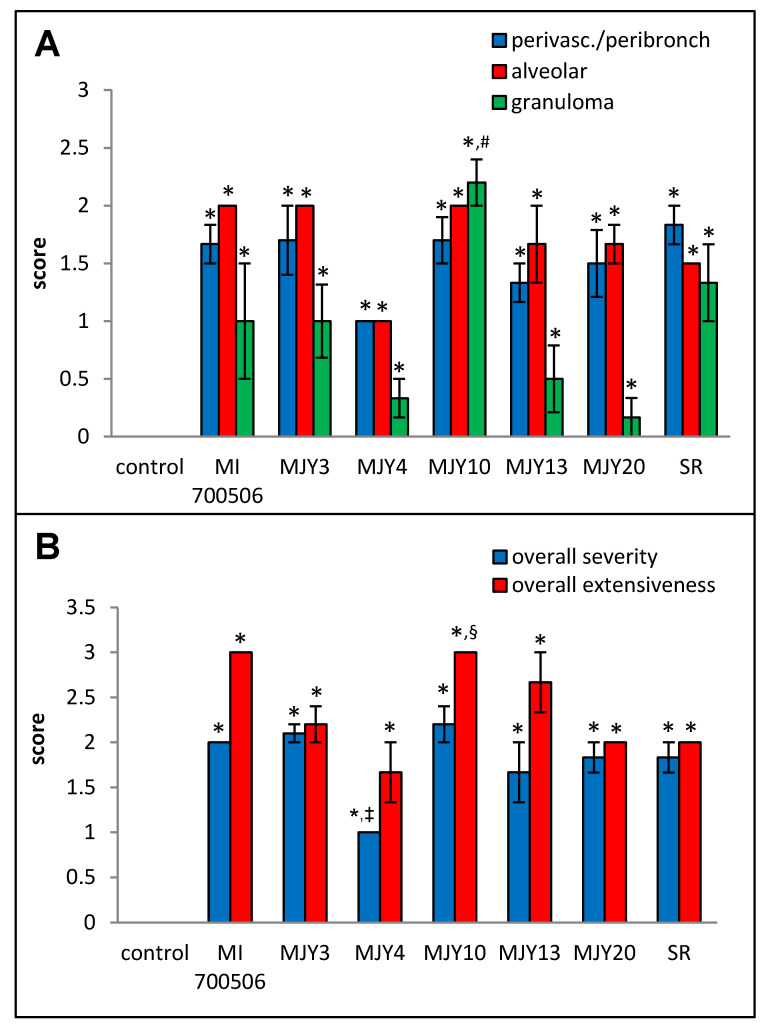
Comparison of individual *M. immunogenum* genotypes for their potential to induce lung histopathological changes in the chronic exposure mouse model. The chronic exposure regimen was based on a repeated dose of 10 µg protein/instillation. Five mice were used for each treatment group. The H&E-stained lung sections were analyzed for overall scores for specific histopathological changes (Panel **A**), as well as scores for severity and extensiveness of damage (Panel **B**), assigned on an arbitrary scale from 0 to 4. * Significantly elevated above control. ^#^ Significantly higher than granuloma scores for MJY4, MJY13, and MJY20. ^‡^ Significantly lower than overall severity scores for MI 700506, MJY3, and MJY10. ^§^ Significantly higher than overall extensiveness scores for MJY3, MJY4, and MJY20.

**Figure 9:**
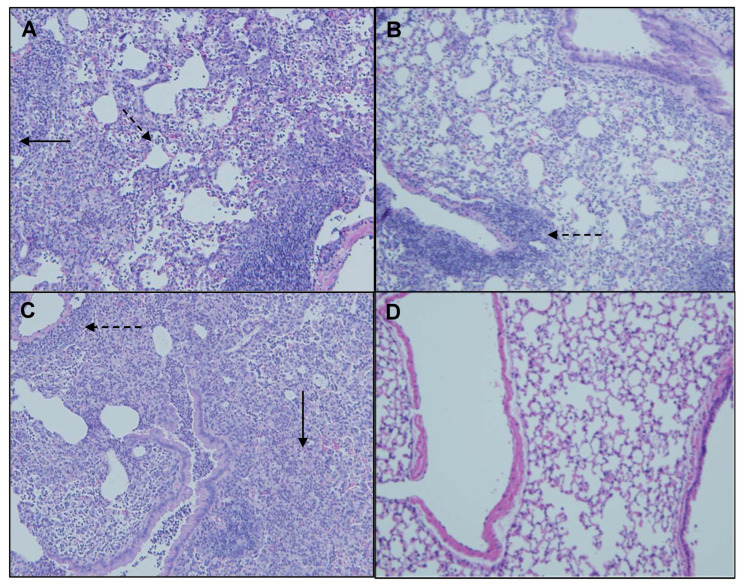
Representative H&E-stained lung sections from mice oropharyngeally instilled with extracts of (**A**) MI 700506, (**B**) MJY4, (**C**), MJY10, and (**D**) endotoxin-free PBS. Five mice were used for each treatment group. Magnification 10×. Perivascular primary lymphocyte infiltration (dashed arrows) and granulomas (solid arrows).

Histopathological examination of H&E-stained lung sections showed mild to moderate perivascular/peribronchial infiltration and moderate alveolar infiltration with no significant differences between the genotype groups. Granuloma formation, however, was significantly more severe in the MJY10 group compared to MJY4, MJY13, and MJY20, the groups with the lowest scores for granuloma formation (Figure 8 and Figure 9). Overall severity scores were significantly lower for MJY4 than for MI 700506, MJY3, and MJY10, and overall extensiveness scores were significantly higher for MJY10 than scores for MJY3, MJY4, and MJY20.

### 2.5. Co-Exposure with MI 700506 and P. fluorescens

**Figure 10:**
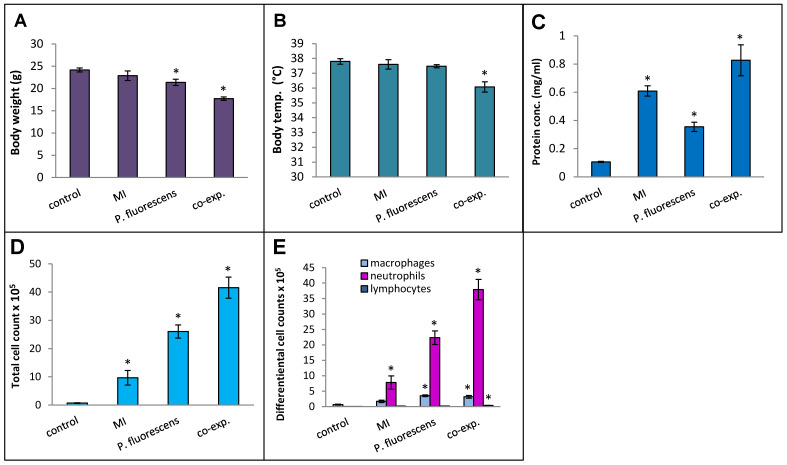
Effect of co-exposure of mouse lung with *M. immunogenum* 700506 (“MI”) and the Gram-negative co-contaminant *Pseudomonas fluorescens* (Pf) on the pathophysiological changes in the chronic exposure mouse model. The mice exposed to the whole cell lysate (6 µg protein of MI alone, 7.8 µg of Pf alone, and 6 µg MI + 7.8 µg Pf) according to the chronic exposure regimen, described in the caption of Figure 1, were sacrificed 4 h after the last instillation. Mice exposed to the PBS vehicle were used as negative control. Four mice were used for each treatment group. Panels: (**A**) Body weight. (**B**) Body temperature. (**C**) Total protein concentration in BAL fluid. (**D**) Total cell count of immune cells in BAL fluid. (**E**) Differential cell count of immune cells in BAL fluid. * Significantly different from control.

**Figure 11:**
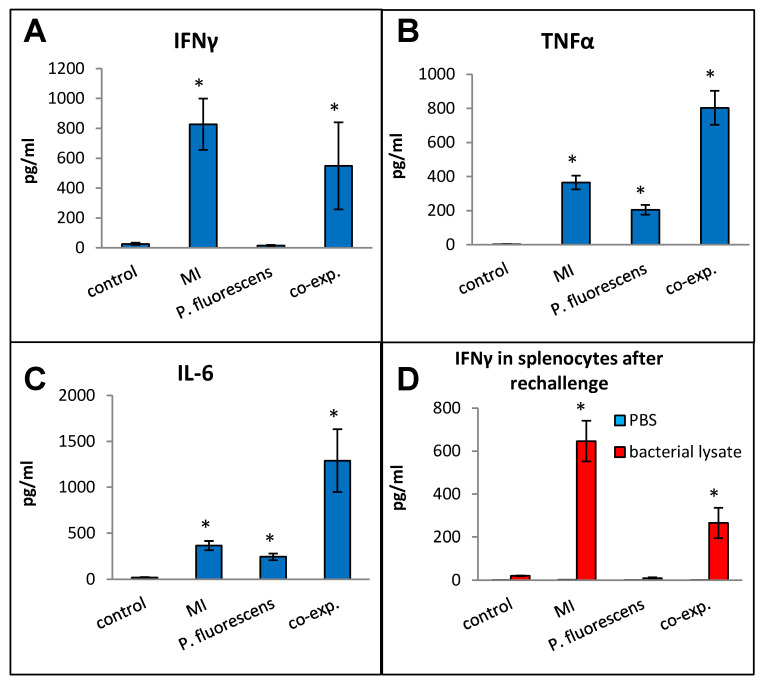
Effect of co-exposure with *M. immunogenum* 700506 (“MI”) and the Gram-negative co-contaminant *Pseudomonas fluorescens* (Pf) on the lung immunological changes and T-cell response in the chronic exposure mouse model. The chronic exposure regimen using the whole cell lysate (6 µg protein of MI alone, 7.8 µg of Pf alone, and 6 µg MI + 7.8 µg Pf). The post-instillation time of sacrifice was the same as described in the caption of Figure 10. Mice exposed to the PBS vehicle were used as negative control. Four mice were used for each treatment group. Panels (**A**–**C**): Immunological changes in BAL fluid, including (**A**) IFN-γ, (**B**) TNF-α, and (**C**) IL-6. Panel (**D**): Ex-vivo T-cell response (measured as release of IFN-γ) in the rechallenged splenocytes derived from the corresponding mice treated with a given antigen type (MI, Pf, or MI + Pf). * Significantly elevated above control.

*Pseudomonas* species are frequently found in *M. immunogenum*-contaminated MWFs in industrial operations; so, in order to assess the effects of simultaneous exposure to these two microbial types, C57BL/6J mice were instilled with whole lysate proteins derived from equivalent cell counts (2.4 × 10^7^ cfu) of each; this corresponded to a dose of 6 µg of MI 700506 lysate protein and 7.8 µg of *P. fluorescens* cell lysate protein. The mice were instilled according to the 3-week chronic exposure regimen used in this study and sacrificed 4 h after instillation of the final dose. The co-exposed mice showed signs of dyspnea, and both body temperature and body weight at the time of sacrifice were lower compared to the control mice and mice instilled with either 6 µg MI 700506 cell lysate protein or 7.8 µg *P. fluorescens* lysate protein (Figure 10A,B). Protein infiltration in the exposed lung was significantly higher for MI 700506 than *P. fluorescens* and the co-exposure showed an additive effect (Figure 10C). The total cell count in BAL fluid for the *P. fluorescens* group was significantly higher than that for the MI 700506 group, and the count for the co-exposure group was approximately additive (Figure 10D). Most of this effect seemed to come from neutrophil infiltration (Figure 10E). In terms of cytokine response in the BAL fluid (Figure 11A–C), MI showed higher induction of proinflammatory cytokines, particularly IFN-γ and TNFα as compared to *P. fluorescens*, though it varied in a cytokine-dependent manner. However, co-exposure showed a synergistic effect on TNFα and IL-6 levels.

Splenocytes from exposed mice were cultured and each group was rechallenged with the same antigen that was used for in vivo exposure (Figure 11D). The rechallenged splenocytes from the control mice and mice exposed to *P. fluorescens* released very little IFN-γ, whereas those from mice exposed to MI 700506 showed a strong IFN-γ response. Interestingly, the IFN-γ response from co-exposed mice was significantly lower than that from mice exposed to MI 700506 alone.

**Figure 12:**
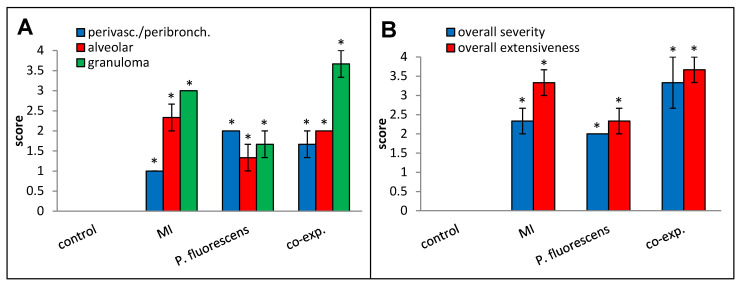
Effect of co-exposure to *M. immunogenum* 700506 (“MI”) and *Pseudomonas fluorescens* cell lysates on lung histopathological changes. Four mice were used for each treatment group. H&E-stained lung sections were scored for the following changes on a scale from 0 to 4 as described in the Materials and Methods section: (**A**) Perivascular/peribronchial infiltration, alveolar infiltration, and granuloma formation. (**B**) Overall severity and extensiveness. * Significantly elevated above control.

Perivascular/peribronchial infiltration, granuloma formation, overall histopathological severity, and overall histopathological extensiveness all trended higher in the co-exposed mice compared to the mice exposed to MI 700506 alone (Figure 12).

## 3. Discussion

Chronic HP is a serious and often progressive environmental or occupational disease that is caused by the inhalation of small, mostly organic antigens. Diagnosis is complicated by similarities to other interstitial lung diseases and the fact that the specific etiology and pathogenesis mechanisms of HP still are not well understood [7].

Animal models of HP have been useful in furthering the knowledge about the cellular and molecular mechanisms involved in the different forms of HP, and the most common mouse model is based on the use of whole cell lysates of *S. rectivirgula*, the antigen associated with the HP form called Farmer’s lung. HP related to the occupational use of MWFs is often associated with the inhalation of aerosols containing *M. immunogenum*, and two recent peer-reviewed reports present findings of HP-like phenotypes in mice exposed to *M. immunogenum* using three-week exposure regimens [57,58]. This present study was designed to further develop the *M. immunogenum* HP model by using a method that offers both consistent dose delivery as well as non-invasiveness and determining an optimal dose of antigen and timepoint of sacrifice to capture the spectrum of immunological and histopathological responses associated with the HP phenotype. While previous HP models have mainly relied on intranasal or intratracheal instillation of the antigen, in this study, we instilled the mice oropharyngeally. Our preliminary experiments showed that the oropharyngeal method resulted in a very reproducible deposition of >90% of the instilled material in the lungs, whereas deposition of intranasally instilled material was highly variable and usually <20%. Furthermore, previous studies have shown that, compared with intratracheal instillation, oropharyngeal instillation results in a more even distribution of deposited material in the lungs and is less invasive [61].

### 3.1. Dose–Response Relationship and Optimal Dose Selection

A linear dose–response relationship was observed for the majority of the immunological parameters in the exposed lung. However, the presentation of lung pathology showed heterogeneity or non-significant increases with doses due to inter-animal variations; this is consistent with pathological heterogeneity in human HP cases [62]. In terms of selecting an appropriate dose that induces a distinct HP-like pathology without necrotizing lung injury, a 10 µg mycobacterial protein dose was considered optimal for further studies.

### 3.2. Capturing the HP-Specific Response Post-Exposure

Chronic HP is an adaptive cell-mediated immune disorder characterized by Th1 immune response and alveolar lymphocytosis [63] leading to an increased number of Th1 cytokines as well as lymphocytes in BAL fluid. The majority of the Th1 cytokines peaked at 4 h post-exposure (after the final dose instillation in the exposure regimen), as did also the infiltration of serum proteins and neutrophils. While the initial increase in neutrophils rapidly abated from 4 h to 48 h, the number of lymphocytes showed a relatively strong increase at the 48 h timepoint to make up 25% of the total number of immune cells in the lung. This is broadly consistent with clinical observations of an initial neutrophil influx in the lung (measured in BAL fluid) immediately after exposure, followed by an increase in the percentage of lymphocytes up to 30% to 70% after 24–48 h [64]. Similar observations have also been made in experimental HP in *S. rectivirgula*-exposed mice [56,65]. Taken together, a 4 h timepoint post-exposure in this model appeared to be the most logical end point to capture both the Th1 cytokine changes as well as pathological changes consistent with a chronic HP-like immunophenotype, whereas a later timepoint (48 h) appeared to be ideal for capturing the lymphocytic events.

### 3.3. Effect of Whole Cells vs. Cell Lysates in Mycobacterial Induction of HP

Most mouse models of HP have relied on bacterial cell lysates as antigens. Given the harsh environment of MWFs and the shear forces involved in the spraying processes, it can be expected that much of the microbial contamination consists of non-viable bacteria and bacterial cell fragments or cell constituents, which can be highly immunogenic despite the lack of viability. Contaminating microorganisms are, however, routinely isolated by culturing, and cfu counts per ml are often quite high. Taken together, this implies the prevalence of mycobacteria in MWF as both live cells and dead cells/cell lysates depending on the prevailing conditions. The cellular and molecular context in which an antigen is presented to the immune system can influence the immune response [66,67]. In a comparison between the responses to whole cell lysates and the equivalent amount of monodispersed cell suspensions of *M. immunogenum*, we found that the latter (cell suspensions) elicited a generally more severe phenotype. The highest dose of monodispersed cell suspension, 5 × 10^6^, caused evident dyspnea, strong increases in BAL fluid parameters, and moderate overall histopathological severity. The equivalent amount of total mycobacterial protein in the form of whole cell lysate, 1.2 µg, yielded a considerably milder immunological response and mild overall histopathological severity. Although care was taken during the preparation of the lysate to avoid temperature increases, the unavoidable shearing and foaming during the bead beating process may have damaged some protein antigens, which is one possible explanation for the results. It is also possible that infectious processes may contribute to the total immune response when using viable cells. Preliminary results from our laboratory, however, have indicated that viable *M. immunogenum* cells are cleared rapidly from the lung after oropharyngeal instillation.

HP is considered to be a Th1-driven disease. Several studies using chronic HP models, however, have found either none or only mild increases in IFN-γ and IL-12 levels after antigen exposure [56,68,69]. Despite the fairly low levels of IFN-γ in BAL fluid at all post-exposure timepoints in this study, splenocytes from exposed animals released robust amounts of IFN-γ after rechallenging with the same antigen. Schuyler et al. [70] used Th1 and Th2 cell lines from lung-associated lymph nodes of *S. rectivirgula*-sensitized mice to show that only Th1 cells could adoptively transfer HP to recipients, and the ability to induce HP was associated with the amount of IFN-γ released by antigen-stimulated cells. Other studies using the *S. rectivirgula* model have demonstrated the role of Th1 lymphocytes in HP by showing the requirement of IFN-γ for the development of the HP phenotype [71]. Our results provide further evidence for the role of Th1 cells in HP development and extend this observation to *M. immunogenum*-induced HP. It is worth noting that the amounts of IFN-γ released after rechallenging splenocytes with the different *M. immunogenum* genotypes correlated well with most BAL fluid parameters as well as granuloma formation 4 h after final dose instillation in the exposure regimen. Recent reports have presented clinical and experimental evidence of Th2 polarization in chronic HP and a role for Th2 cells in the development of fibrosis [72,73,74]. We did not, however, detect any increase in the levels of the Th2 cytokine IL-4 in the BAL fluid of exposed mice, and there was no IL-4 response in rechallenged splenocytes.

### 3.4. Effect of Co-Exposure on Induction of Mycobacterial HP

While *M. immunogenum* has been most often implicated as the etiological agent in MWF-associated HP outbreaks, Gram-negative bacteria, most notably *Pseudomonas* spp., have also been isolated from HP-associated MWFs. Endotoxin from the cell walls of Gram-negative bacteria is strongly immunogenic. Low doses of endotoxin have been associated with both protective and risk-promoting effects on asthma, while inhalation of larger amounts can result in acute airway inflammation, and there is evidence of a switch from a Th2-driven eosinophilic response at low-dose exposure to Th1-driven neutrophilic inflammation associated with exposure to larger doses [75]. Exposure to *Pseudomonas* endotoxin may also skew the polarization of conventional dendritic cells (cDCs) in the lung in favor of neutrophilic response [76]. Contaminating endotoxin in MWFs has also been implicated in exacerbating MWF-associated HP [57,58,77]. Since *M. immunogenum* and *P. fluorescens* have been isolated simultaneously from used MWF, we investigated the effect of the co-exposure of mice to these two microorganisms on the development of HP immunopathology in the chronic HP model. We found that based on most parameters in BAL fluid, including protein concentration, total number of cells, number of neutrophils, TNFα levels, and IL-6 levels, the inflammatory response was significantly stronger in co-exposed mice compared to mice exposed to *M. immunogenum* alone. In contrast, the IFN-γ response was weaker for the co-exposed mice than for mice exposed to *M. immunogenum* alone and the difference was significant in rechallenged splenocyte T-cell response, unlike the BAL fluid. Collectively, the IFN-γ data from the lung and spleen indicated that the Th1 immune response to MI is suppressed by the Gram-negative co-contaminant *P. fluorescens*.

These results suggest that the observed more severe reaction and worse general condition of co-exposed mice were not associated with the Th1 cell-mediated response. An earlier study [58] compared mice exposed to MWF spiked with *M. immunogenum* and endotoxin and mice exposed to *M. immunogenum* in MWF alone and found significantly more severe lung lesions, slightly higher numbers of macrophages and neutrophils in BAL fluid, and higher BAL fluid levels of IL-10 in the co-exposed mice. Our results are in part consistent with their findings, but comparisons are complicated by the much later timepoint of sacrifice, 96 h after the last exposure, used in their study. Furthermore, the difference in instillation methods, nasal vs. oropharyngeal, is likely to result in differences in the distribution of antigens in the lung.

## 4. Materials and Methods

### 4.1. Animals

Six-week-old male C57BL/6J mice from the Jackson Laboratory (Bar Harbor, ME, USA) were used for this study. The mice were housed in ventilated PIV cages under pathogen-free conditions and provided standard rodent chow and water ad libitum. The protocols and procedures were approved by the Institutional Animal Care and Use Committee of the University of Cincinnati.

### 4.2. Bacterial Strains/Genotypes and Culture Conditions

*Mycobacterium immunogenum* ATCC strain 700506 (abbreviated as MI 700506), originally isolated from machinist’s HP-associated metalworking fluids [39], was used for the optimization experiments in the current study. Six other genotypes/strains of this species, isolated in our laboratory based on an industry-wide screening of field-collected used metalworking fluids [53], were used for the genotype comparison studies using MI 700506 as a reference strain. All strains were maintained by subculturing on Middlebrook 7H10 agar (BD Diagnostics, Sparks, MD, USA) supplemented with 10% Oleic acid–Albumin–Dextrose–Catalase (OADC) enrichment (BD Diagnostics, Sparks, MD, USA).

*Pseudomonas fluorescens* (ATCC 13525) was used as a representative species for Pseudomonads, which are abundant Gram-negative co-contaminants in mycobacteria-colonized MWF. This organism was subcultured at 30 °C using trypticase soy broth.

### 4.3. Preparation of M. immunogenum (MI) Cell Lysate for Animal Dosing

*M. immunogenum* (MI) strains were grown in Middlebrook 7H9 broth (BD Diagnostics, Sparks, MD, USA) supplemented with 10% Oleic acid–Albumin–Dextrose–Catalase (OADC) enrichment (BD Diagnostics, Sparks, MD, USA) by continuous shaking at 200 rpm. MI 700506 was grown at 37 °C, and all other MI strains/genotypes were grown at 30 °C. When the cultures had reached 120 Klett Reading, after approximately 48–72 h, as measured in a Klett photoelectric colorimeter (Klett, New York, NY, USA), cells were harvested by centrifugation at 3000× *g*, and the pellet washed three times with endotoxin-free phosphate-buffered saline (PBS). Cells were resuspended in endotoxin-free PBS. One-ml cell suspension and 0.4 g sterile 0.1 mm glass beads were combined in a sterile 2 mL tube, and the cells were lysed by bead beating (4 × 1 min) in a Mini-Bead beater (Biospec Products, Bartlesville, OK, USA) with cooling on ice in between. The lysate was aspirated off and stored at −80 °C until further use. The protein concentration was determined using the DC™ Protein Assay from BioRad (Hercules, CA, USA). One µg total mycobacterial protein was equivalent to 4.2 × 10^6^ cfu, using a monocellular bacterial suspension of MI prepared as described below. The challenge dose range was 0–30 µg MI protein/mouse. For the co-exposure studies, *P. fluorescens* cell lysate was prepared using the same approach as employed for MI.

### 4.4. Preparation of Saccharopolyspora rectivirgula (SR) Lysate

*Saccharopolyspora rectivirgula* (SR), strain A1313 (ATCC 29034), a thermoactinomycete bacterium known to induce Farmer’s HP in mice, was grown in trypticase soy broth at 55 °C with continuous shaking at 200 rpm until the culture density had reached 120 Klett Reading measured as described above. The cells were separated by centrifugation and washed three times with endotoxin-free PBS. The final cell pellet was resuspended in endotoxin-free PBS, and the cells were lysed by sonication on ice. An aliquot was dried at 80 °C to obtain total dry weight/mL lysate, and the remainder of the lysate was stored at −80 °C until further use. The challenge dose was 250 µg (dry weight basis)/mouse.

### 4.5. Preparation of Monocellular Mycobacterial Suspensions

Monocellular suspensions of *M. immunogenum* were prepared, forcing the bacterial suspension through a 26-gauge needle 20 times and centrifuging the resulting suspension at 100× *g* for 5 min to remove remaining clumps. Bacterial count (cfu per ml) was determined by spread plating serial dilutions of the monocellular suspension on Sauton’s agar. The doses evaluated were 10^5^, 10^6^, and 5 × 10^6^ cfu per mouse.

### 4.6. Animal Exposure Protocol

After light anesthesia with isoflurane, mice were exposed to HP antigen preparation (MI or SR) using oropharyngeal route [61]. Five mice per treatment group were used with some exceptions due to unanticipated losses as specified in figure legends. Briefly, a 50 µL inoculum (containing the desired dose level) was placed at the back of the tongue while holding the tongue. The nose was then held until the animal had fully aspirated the inoculum and drawn two deep recovery breaths. *S. rectivirgula* (SR) lysate was used as a positive control for the HP phenotype, with a dose of 250 µg (total dry weight) per instillation. Instillations were performed on three consecutive days per week for three weeks. Unless otherwise indicated, mice were sacrificed 4 h after the last instillation.

### 4.7. Bronchoalveolar Lavage and Analyses

After intraperitoneal injection with Euthasol^®^, the lungs were lavaged with 2 × 1 mL PBS, and cells were spun down at 300× *g*, 4 °C, for 10 min. The supernatants were combined and stored at −80 °C until further use. Cells were resuspended in PBS containing 5% fetal bovine serum (FBS) and counted using a hemocytometer to obtain the total number of cells per ml of BAL fluid. Differential cell counts in BAL fluid were obtained by counting >300 cells on slides prepared using Cytospin (Cytospin3; Shandon Scientific Ltd., Runcorn, Cheshire, UK) and stained using PROTOCOL Hema 3 staining kit (Fisher Scientific, Waltham, MA, USA).

BAL fluid supernatants were analyzed for total protein concentration using the DC™ protein assay from BioRad (Hercules, CA, USA), and for concentrations of cytokines using Ready-Set-Go!^®^ ELISA kits from eBioscience (San Diego, CA, USA) according to the manufacturer’s instructions.

### 4.8. Histopathological Analyses

After bronchoalveolar lavage, the lungs were formalin-fixed, and H&E-stained tissue slides were prepared by the institutional histopathology core facility. Histopathological analysis was performed on three random slides from each treatment group. Peribronchial/perivascular infiltration, alveolar infiltration, granuloma formation, and overall severity were scored as follows: 0 = no inflammation; 0.5 = very mild; 1 = mild; 2 = moderate; 3 = severe; 4 = very severe. the overall extensiveness of lung involvement was scored as follows: 0 = no inflammation; 1 = 0–10%; 2 = 10–30%; 3 = 30–50%; 4 = >50%.

### 4.9. Splenocyte T-Cell Response Analysis

At the time of sacrifice, the spleens of the challenged mice were removed antiseptically and splenocytes were isolated. Briefly, spleens were pushed through a 100 µm cell strainer into Hank’s balanced salt solution (HBSS). After collecting dissociated cells at 300× *g*, 4 °C, for 8 min, the pellet was resuspended in RPMI 1640 containing 2 mM L-glutamine, 100 U/mL penicillin/streptomycin, 10% fetal calf serum, and 50 µM β-mercaptoethanol. The splenocyte T-cells were plated in complete RPMI 1640 medium, and the cells from each mouse were rechallenged with the mycobacterial protein extract originally used for treatment of that animal, using 20 µg mycobacterial protein per ml culture medium. PBS was added to control wells. After 48 h, the medium was collected and frozen at −80 °C. The levels of IFN-γ and IL-4 in the medium were measured by ELISA using commercial kits (eBioscience, San Diego, CA, USA) after the removal of cells and solid particles by centrifugation.

### 4.10. Statistical Analyses

Statistical analysis was performed using SigmaStat software version 15.0 (SPSS Inc., Chicago, IL, USA). Group means were compared by one-way analysis of variance, followed by the Holm–Sidak post hoc test for significance. Log transformation of variables was performed when necessary to obtain normal distribution. Data were reported as means ± S.E. *p* values < 0.05 were considered statistically significant.

## 5. Conclusions

This study led to the optimization of an improved mouse model of chronic HP caused by *M. immunogenum*. Specifically, the optimization experiments based on the oropharyngeal route of dose instillation helped clarify the role of MI’s antigen dose, the form of antigen (cell lysates vs. live cells), and post-instillation timepoint of sacrifice for the chronic HP mouse model. An estimated dose of 10 µg MI protein per animal was considered optimal as it induced a distinct HP-like pathology without necrotizing lung injury. Within a common equivalent dose range (in terms of protein content), MI live cell suspensions caused a more severe phenotype as compared to MI cell lysates. A post-exposure time-point of 4 h for mice harvest was found to be optimal for capturing the peak Th1 immune response and histological changes consistent with HP pathology; however, a 48 h timepoint seemed ideal for capturing the lymphocytic events. The results confirmed the importance of Th1 lymphocytes and their Th1 response in the *M. immunogenum*-induced HP pathology while elucidating the interfering effect of co-exposure to Pseudomonas (a dominant Gram-negative co-contaminant that serves a source of endotoxin) in the Th1 response. However, unlike the observed interference in adaptive immune response, Pseudomonas co-exposure exacerbated the innate immune response; this might explain the observed more severe reaction and worse general condition of the co-exposed mice. The use of the optimized chronic HP mouse model enabled the meaningful comparison of the field MWF-isolated MI strains/genotypes. Different MI genotypes showed significantly different immunogenicity and HP-inducing potential, thereby allowing for the identification of a polar pair of immunogenic strains. MJY10 (or MI 700506) was identified as the most immunogenic, and MJY4 (or MJY13) as the least immunogenic genotype. This identified strain pair will facilitate future efforts to identify HP causal antigens and their epitopes critical in the development and diagnosis of *M. immunogenum*-induced HP. The causal antigens that show selective reactivity with HP patients (symptomatic) but not with the MWF-exposed control subjects (asymptomatic) could be identified based on functional screening using the optimized HP mouse model and follow-up validation using clinical specimens. These developments could pave the way for designing differential immunodiagnosis assays for machinist’s HP. Taken together, the optimized model and the immunogenically polar MI strains pair are expected to facilitate future studies on (1) the identification of causal antigens critical for immunodiagnostics and (2) the hitherto unclear pathogenesis mechanisms and therapeutic targets for prevention and treatment of this interstitial lung disease.

## Data Availability

Data is contained within the article.
